# Common Side Effects of Pfizer COVID-19 Vaccine: An Experience From Pakistan

**DOI:** 10.7759/cureus.40878

**Published:** 2023-06-23

**Authors:** Syed Muhammad Safi Haider, Shaf Ali Alvi, Hamza Khan, Rameen Majeed, Tatheer Syed, Adnan Anwar, Atif A Hashmi

**Affiliations:** 1 Internal Medicine, Hamdard College of Medicine and Dentistry, Karachi, PAK; 2 General Surgery, Hamdard College of Medicine and Dentistry, Karachi, PAK; 3 Biochemistry, Jinnah Sindh Medical University, Karachi, PAK; 4 Public Health, Jinnah Sindh Medical University, Karachi, PAK; 5 Physiology, Hamdard College of Medicine and Dentistry, Karachi, PAK; 6 Internal Medicine, Essa General Hospital, Karachi, PAK; 7 Pathology, Liaquat National Hospital and Medical College, Karachi, PAK

**Keywords:** side effects, vaccine, covid-19, joint pain, swelling, pain, fever, burning, pfizer vaccine

## Abstract

Introduction

The severe acute respiratory syndrome coronavirus 2 (SARS‑CoV‑2) epidemic spread quickly. Vaccines are now being distributed to stop the infectious spread and halt fatalities. The Pfizer-BioNTech vaccine was the first mRNA-based vaccine introduced to boost immunity against COVID-19; however, it could lead to various adverse reactions. Therefore, the aim of this study was to assess the prevalence of Pfizer vaccine side effects among participants.

Methods

This was a multicenter cross-sectional study that was performed using a non-probability sampling method. The study period was about six months from March 1, 2022, to August 31, 2022. A total of 1000 participants who received two doses of the Pfizer vaccine met the inclusion criteria. Demographic details of participants, for example, gender, age, comorbidities, Pfizer vaccine with both doses along with booster dose, previous exposure to coronavirus disease 2019 (COVID-19) infection, and the incidence of any local and systemic side effects following the first and second doses of vaccine, were reported.

Results

The study findings showed that out of 1000 participants, 644 (64.4%) were males and 356 (35.6%) were females; their mean age was 43.06±14.98 years. Among them, 280 (28.0%) had hypertension and 356 (35.6%) had diabetes. Following the first dose of the Pfizer vaccine, burning at the injection site and fever were the most commonly reported side effects in 704 (70.4%) and 700 (70.0%) participants, respectively. Following the second dose of the Pfizer vaccine, muscle pain was the most commonly reported side effect in 628 (62.8%) participants.

Conclusion

This study concluded that the most frequent adverse effects of the Pfizer vaccine were burning at the injection site, fever, pain at the injection site, muscle pain, swelling at the injection site, and joint pain. Moreover, the first dose was associated with more side effects than the second dose.

## Introduction

The novel coronavirus disease 2019 (COVID-19) was recognized in 2019. Globally, it spread rapidly and had a high fatality and morbidity rate. As a consequence, the World Health Organization considered severe acute respiratory syndrome coronavirus 2 (SARS-CoV-2) as a pandemic in March 2020 [1,2]. SARS-CoV-2 infections can cause a wide spectrum of symptoms, from mild or asymptomatic infections to serious pulmonary and multi-organ illnesses that are fatal [3,4]. Additionally, novel SARS-CoV-2 variants have emerged because of the high rates of transmission, creating a dilemma in controlling this pandemic [5,6]. Even though governments and organizations all around the world had taken several steps to stop the pandemic spread, the only option for ending the imminent danger was to develop a vaccine [7].

AstraZeneca, Janssen (Johnson & Johnson), Sinopharm, Sinovac, Sputnik V, and Pfizer BioNTech were a few COVID-19 vaccines to fight the epidemic [8,9]. The effectiveness of these vaccines in avoiding COVID-19 infection varies, but each form of immunization has specific advantages and disadvantages in terms of efficacy, immunogenicity, and effectiveness [10]. The Pfizer-BioNTech COVID-19 vaccine was approved by the US Food and Drug Administration (FDA) for emergency use on December 11, 2020 [11].

The Pfizer-BioNTech (BNT162b2) vaccines depend on mRNA technology. The coronavirus has an S protein, which is a spike-like surface characteristic [12-14]. mRNA technology is a novel technique that has recently been created for potential use in vaccine production, and several are currently being tried [15]. The Pfizer-BioNTech vaccine is considered the first mRNA-based immunization for infectious diseases approved for use in humans [15].

In addition to numerous local side effects like redness, pain, and swelling at the injection site, Pfizer-BioNTech company has also reported numerous systemic reactions like headache, fever, fatigue, chills, diarrhea, vomiting, and deteriorating muscle/joint pain. Additionally, serious deleterious events such as appendicitides, allergic reactions, acute myocardial infarctions, and cerebrovascular problems have been documented [16]. Furthermore, postmarketing research revealed a minor variation in the frequency and types of side effects described by vaccine recipients [17,18]. Negative outcomes should remain closely observed as vaccine distribution rises worldwide. mRNA technology used in the Pfizer vaccine is novel, so it is still difficult to anticipate every side effect [19].

The safety of COVID-19 immunization is of utmost importance to guarantee that the benefits outweigh the risks. However, because of the small sample size, inclusion requirements, and subject traits, which may vary from the community receiving the vaccination, serious or uncommon adverse events may not be discovered in phase 3 trials [20]. To identify long-term and uncommon adverse events linked to the vaccine, the WHO advised post-marketing assessment of the safety profile of all vaccines [21].

A dearth of independent research on the safety of vaccines could have an adverse impact on vaccine uptake, which needs to be hastened in the coming months in order to break the virus and its numerous variants out of this vicious cycle [22]. Therefore, this study aimed to assess the Pfizer vaccine side effects among participants in the Pakistani population.

## Materials and methods

This multicenter cross-sectional study was conducted using a non-probability sampling method. The study period was about six months from March 1, 2022 to August 31, 2022. Ethical approval was obtained from Essa General Hospital (Essa/75/2022) before conducting the study. The data were obtained prospectively. A total of 1000 participants who received both the first and second doses of the Pfizer vaccine met the inclusion criteria. All participants were above 18 years of age. Participants who had received a vaccination with a different vaccine (other than Pfizer) or who had never received a COVID-19 vaccination were excluded from the study. Immunosuppressed patients, those with active COVID-19 infection, and those undergoing chemoradiation for malignancies were excluded from the study.

The objectives of the study were briefly explained to each participant, and they were then requested their informed permission before beginning the questionnaire. A self-designed questionnaire was used to get the participant's information. Demographic details of participants, for example, gender, age, comorbidities, Pfizer vaccine with both doses along with booster dose, previous exposure with COVID-19 infection, and local and systemic side effects following the first and second doses of vaccine, were reported. Local side effects include pain, swelling, redness, and burning at the site of injection, whereas systemic side effects include fever, chills, headache, shortness of breath, chest pain, nausea, diarrhea, flu-like illness, anxiety, and fatigue. Local side effects also include muscular pain (myalgia), pain in joints, lymphadenopathy, and sore throat. The participants' satisfaction was also documented. 

The data were entered and analyzed using SPSS Statistics for Windows, Version 26.0 (IBM Corp., Armonk, NY, USA). Categorical data were reported as frequencies and percentages, while continuous data such as age, height, weight, and comorbidities were documented as means and standard deviations. 

## Results

A total of 1000 participants vaccinated with the Pfizer vaccine were involved in the study. There were 644 (64.4%) males and 356 (35.6%) females among them. The mean age of the participants was 43.06±14.98 years. The mean height and weight of the participants were 5.17±0.60 feet and 67.42±15.64 kg, respectively. The mean duration of hypertension and diabetes was 5.45±6.17 years and 3.73±2.79 years, respectively. Out of 1000 participants, 280 (28.0%) had hypertension and 356 (35.6%) had diabetes. Additionally, only 80 (8.0%) participants had previous exposure to COVID-19 infection, as shown in Table 1. 

**Table 1:** The demographic details of vaccinated participants (n=1000) SD, standard deviation; DM, diabetes mellitus; COVID-19, coronavirus disease 2019

Variables		Values
Age (years), Mean±SD		43.06±14.98
Weight (kg), Mean±SD		67.42±15.64
Height (ft), Mean±SD		5.17±0.60
Duration of hypertension (years), Mean±SD		5.45±6.17
Duration of DM (years ), Mean±SD		3.73±2.79
Gender	Male	644(64.4%)
	Female, n(%)	356(35.6%)
Hypertension	Yes, n(%)	280(28.0%)
	No, n(%)	720(72.0%)
Diabetes Mellitus	Yes, n(%)	356(35.6%)
	No, n(%)	644(64.4%)
History of previous COVID-19 infection	Yes, n(%)	80(8.0%)
	No, n(%)	920(92.0%)

**Figure 1:**
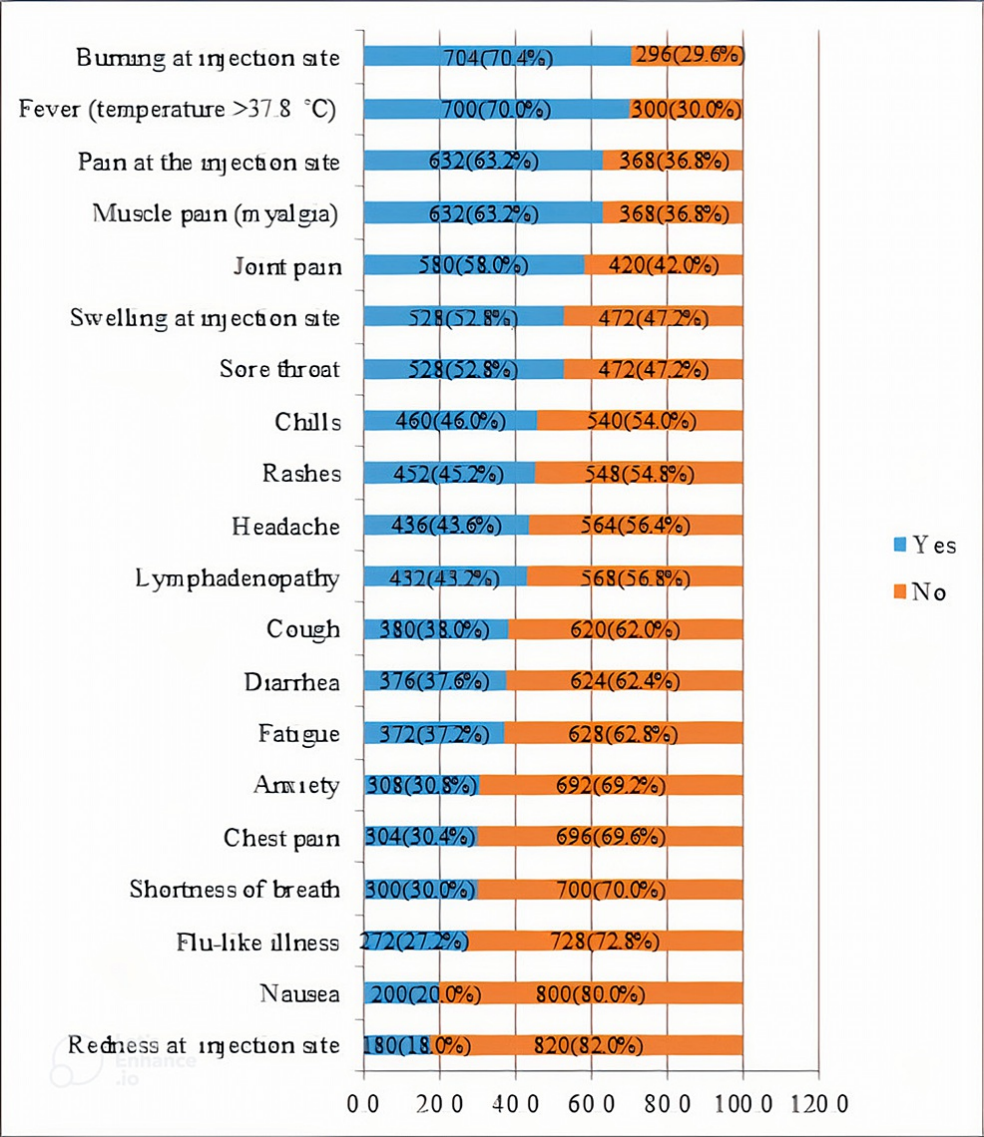
The incidence of side effects after the first dose of the Pfizer vaccine

Following the first dose of the Pfizer vaccine, burning at the site of injection and fever were the most frequently reported side effects in 704 (70.4%) and 700 (70.0%) participants, respectively. Additionally, pain at the injection site in 632 (63.2%) participants followed by muscle pain in 632 (63.2%) recipients was observed. Moreover, other adverse effects such as swelling at the injection site in 528 (52.8%) participants, joint pain in 580 (58.0%) participants, sore throat in 528 (52.8%) participants, and chills in 460 (46.0%) participants were noticed. On the other hand, nausea and redness at the site of injection were the least reported side effects by 200 (20.0%) and 180 (18.0%) participants, respectively, as shown in Figure 1.

**Figure 2:**
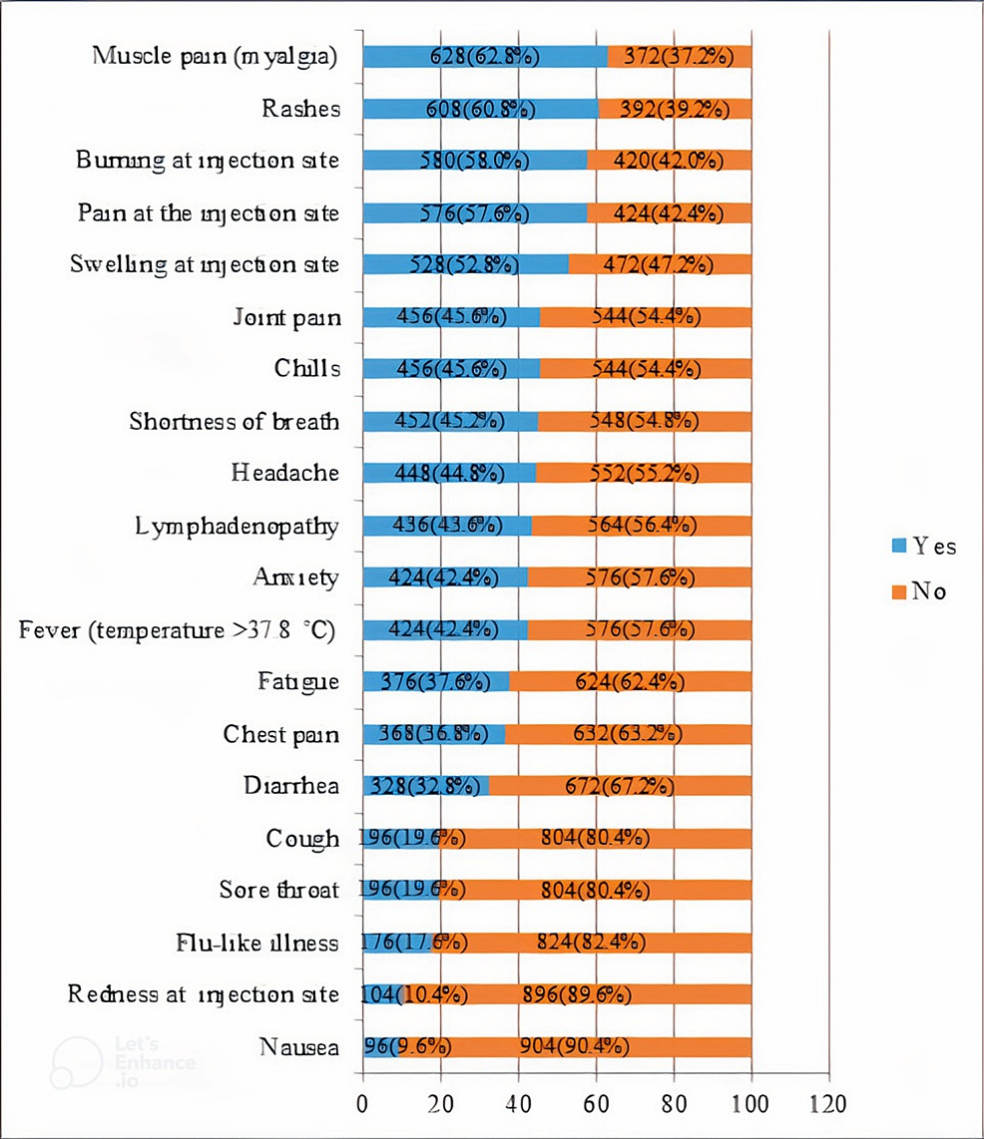
The incidence of side effects after the second dose of the Pfizer vaccine

Following the second dose of the Pfizer vaccine, muscle pain was the most commonly reported side effect in 628 (62.8%) participants, followed by rashes 608 (60.8%) and pain at the injection site 576 (57.6%). Moreover, burning at the injection site 580 (58.0%), swelling at the injection site 528 (52.8%), headache 448 (44.8%), and lymphadenopathy 436 (43.6.6%) were also observed. Likewise, nausea was the least commonly reported side effect by 96 (9.6%) participants receiving the second dose, as shown in Figure 2. 

Table 2 shows the association of the commonest first dose of Pfizer vaccine side effects (fever and pain at the injection site) with age, gender, and comorbidities; a significant association was noted with all these variables (p<0.05), as shown in Table 2.

**Table 2:** The association of commonest side effects of Pfizer vaccine (first dose) with age, gender, and comorbidities *p-value significant as <0.05

Variable		Fever			Pain at the injection site		
		Yes, n(%)	No, n(%)	p-value	Yes, n(%)	No, n(%)	p-value
Age group	<30 years	176(88.0%)	124(62.0%)	<0.001*	76(38.0%)	124(62.0%)	<0.001*
	31-50 years	297(56.6%)	196(37.3%)		329(62.7%)	196(37.3%)	
	>50 years	227(82.5%)	48(17.5%)		227(82.5%)	48(17.5%)	
Gender	Male	400(62.1%)	244(37.0%)	<0.001*	380(59.0%)	264(41.0%)	<0.001*
	Female	300(84.3%)	56(15.7%)		252(70.8%)	104(29.2%)	
Hypertension	Present	180(64.3%)	100(35.7%)	0.017*	208(74.3%)	72(25.7%)	<0.001*
	Absent	520(72.2%)	200(27.8%)		424(58.9%)	296(41.1%)	
Diabetes mellitus	Present	232(65.2%)	124(34.8%)	0.016*	260(73.0%)	96(27.0%)	<0.001*
	Absent	468(72.7%)	176(27.3%)		372(57.8%)	272(42.2%)	

Similarly, the association of second-dose side effects with age, gender, and comorbidities revealed a significant association between age, gender, and diabetes with fever and pain at the injection site (p<0.05), while an insignificant association was found between fever and pain at the injection site with respect to hypertension, (p>0.05), as shown in Table 3.

**Table 3:** The association of commonest side effects of the Pfizer vaccine (second dose) with age, gender, and comorbidities *p-value significant as <0.05

Variable		Fever			Pain at injection site		
		Yes, n(%)	No, n(%)	p-value	Yes, n(%)	No, n(%)	p-value
Age group	<30 years	24(12.0%)	176(88.0%)	<0.001*	152(76.0%)	48(24.0%)	<0.001*
	31-50 years	280(53.3%)	245(46.7%)		272(52.0%)	252(48.0%)	
	>50 years	120(43.6%)	155(56.4%)		151(54.9%)	124(45.1%)	
Gender	Male	296(46.0%)	348(54.0%)	0.003*	344(53.4%)	300(46.6%)	<0.001*
	Female	128(36.0%)	228(64.0%)		232(65.2%)	124(34.8%)	
Hypertension	Present	124(44.3%)	156(55.7%)	0.496	152(54.3%)	128(45.7%)	0.211
	Absent	300(41.7%)	420(58.3%)		424(58.9%)	296(41.1%)	
Diabetes mellitus	Present	200(56.2%)	156(43.8%)	<0.001*	228(64.0%)	128(36.0%)	0.003*

Table 4 compares the incidence of Pfizer vaccine effects after the first and second doses. Except for swelling at the injection site, lymphadenopathy, headache, rashes, anxiety, fatigue, dyspnea, and chest pain, all other side effects significantly reduced after the second dose.

**Table 4:** The comparison of Pfizer vaccine side effects after first and second doses *p-value significant as <0.05

Variable	Side effects after first dose		Side effects after second dose		p-value
	Yes, n(%)	No, n(%)	Yes, n(%)	No, n(%)	
Pain at injection site	632(63.2%)	368(36.8%)	576(57.6%)	424(42.4%)	<0.001*
Swelling at injection site	528(52.8%)	472(47.2%)	528(52.8%)	472(47.2%)	0.462
Redness at injection site	180(18.0%)	820(82.0%)	104(10.4%)	896(89.6%)	<0.001*
Lymphadenopathy	432(43.2%)	568(56.8%)	436(43.6%)	564(56.4%)	<0.001*
Fever (temperature >37.8 ˚C)	700(70.0%)	300(30.0%)	424(42.4%)	576(57.6%)	<0.001*
Headache	436(43.6%)	564(56.4%)	448(44.8%)	552(55.2%)	<0.001*
Nausea	200(20.0%)	800(80.0%)	96(9.6%)	904(90.4%)	<0.001*
Rashes	452(45.2%)	548(54.8%)	608(60.8%)	392(39.2%)	<0.001*
Burning at injection site	704(70.4%)	296(29.6%)	580(58.0%)	420(42.0%)	<0.001*
Flu-like illness	272(27.2%)	728(72.8%)	176(17.6%)	824(82.4%)	<0.001*
Anxiety	308(30.8%)	692(69.2%)	424(42.4%)	576(57.6%)	<0.001*
Muscle pain	632(63.2%)	368(36.8%)	628(62.8%)	372(37.2%)	<0.001*
Fatigue	372(37.2%)	628(62.8%)	376(37.6%)	624(62.4%)	<0.001*
Joint pain	580(58.0%)	420(42.0%)	456(45.6%)	544(54.4%)	<0.001*
Chills	460(46.0%)	540(54.0%)	456(45.6%)	544(54.4%)	<0.001*
Cough	380(38.0%)	620(62.0%)	196(19.6%)	804(80.4%)	<0.001*
Sore throat	528(52.8%)	472(47.2%)	196(19.6%)	804(80.4%)	0.029*
Shortness of breath	300(30.0%)	700(70.0%)	452(45.2%)	548(54.8%)	<0.001*
Diarrhea	376(37.6%)	624(62.4%)	328(32.8%)	672(67.2%)	<0.001*
Chest pain	304(30.4%)	696(69.6%)	368(36.8%)	632(63.2%)	<0.001*

The level of satisfaction with the Pfizer vaccine showed that most participants (n=632, 63.2%) were satisfied and 196 (19.6%) were very satisfied with their vaccination, as shown in Table 5.

**Table 5:** The overall satisfaction with the Pfizer vaccine

Variable		n	%
Overall satisfaction with the vaccine	Very Satisfied	196	19.6
	Satisfied	632	63.2
	No opinion	172	17.2
	Dissatisfied	0	0.0

## Discussion

In this study, we evaluated the side effects of the Pfizer vaccine and found that fever and pain at the injection site were the most common side effects of the Pfizer vaccine. Moreover, most of the side effects significantly reduced after the second dose. 

Any immunization is expected to have temporary side effects owing to the triggered immune response and tissue destruction at the injection site. The side effects were categorized as localized or systemic [23]. Therefore, this study demonstrated the reported local and systemic side effects after receiving the Pfizer vaccine among recipients.

Cross-sectional research was performed in the Czech Republic on the adverse events experienced by healthcare professionals after receiving the COVID-19 vaccine [24]. In this study, there were 522 participants, of whom 77% were women and 55.7% were between the ages of 31 and 54 years. Pain at the injection site (85.2%), swelling at the injection site (10.2%), and redness at the injection site (8.4%) were the most commonly experienced local adverse effects. The most frequently mentioned systemic side effect was fatigue (54.2%), which was then followed by headaches (34.3%), myalgia (28.4%), and chills (26.4%) [24]. 

Similarly, a systematic review revealed that the most common local side effect was localized swelling (33.57%), followed by injection site pain (77.34%), which was thought to be the most frequent local side effect [25]. These results did not line up with another study by Elnaem et al, who found that among people who received the Pfizer vaccination, pain (61.1%) and fatigue (48.8%) were frequently noticed side effects [26]. 

Likewise, another study demonstrated that systemic side effects were fatigue (43%), which was the most frequent systemic adverse effect, followed by muscular pain (39.67%), headaches (33.27%), pain in joints (25.75%), fever (18.0%), chills (18.34%), itching (9.38%), swelling of lymph node (7.86%), vomiting (7.58%), difficulty in breathing (7.86%), and diarrhea (6.36%), among other symptoms [25]. These were widespread and consistent with the research by Alghamdi et al., who observed that the typical side effects of the vaccination were lethargy, fever, and headaches [27]. Another study found that headaches and tiredness were the two adverse effects that were noted the second and third most frequently [28]. 

Interestingly, another study found that adverse events were more prevalent after the second dosage than they were after the first dose. After the first dosage, there were 79% adverse effects, and 84% after the second [25]. According to US FDA research relating both dosages of the vaccine, the frequency of local adverse effects was higher after the second dose [28]. Abu-Hammad et al. showed that after the second dosage, harmful effects were more common [29]. Elnaem et al. found that about 40% of side effects were more frequent after the second dose, predominantly in people who received the Pfizer vaccine as opposed to those who received the AstraZeneca or Sinovac vaccine [26]. As far as the present study is concerned, local and systemic side effects were greater after the first dose than the second dose. Table 6 compares the findings of our study with the existing literature.

**Table 6:** Comparison of our study findings with existing literature N/M, not mentioned; N/A, not applicable

Authors	Commonest side effects of Pfizer vaccine					
	Fever	Pain at injection site	Myalgia and fatigue	Swelling at the injection site	Redness at injection site	Reference
Riad et al.	N/M	85.2%	28.4% and 54.2%	10.2%	8.4%	24
Dighriri et al.	18.0%	77.34%	39.67% and 43%	33.57%	N/M	25
Elnaem et al.	N/M	61.1%	48.8%	N/M	N/M	26
Alghamdi et al.	42%	N/M	49.8%	N/M	N/M	27
Our study	70%	63.2%	63.2% and 37.2%	52.8%	18%	N/A

Apart from Pfizer, Moderna was the other most common mRNA-based COVID-19 vaccine. Many studies compared the adverse effects of these vaccines, and the side effects of these two vaccines are comparable [30].

Limitations of the study

This study had a few limitations. This was a self-reported study based on participant experiences with side effects that were not clinically evaluated or confirmed and could be associated with other aspects apart from the vaccine; as a result, this study was unable to determine the causality of serious events as recommended by the WHO. Further investigation and studies are needed to identify serious side effects and demonstrate a clear causative link. Moreover, the study was limited to a few hospitals and vaccination centers, and all the vaccination facilities were not covered. Additionally, the long-term consequences of vaccine and comparison with vaccine-induced immunity vs. natural immunity after infection was not compared in our study. 

## Conclusions

This study concluded that the most frequent side effects of the Pfizer-BioNTech COVID-19 vaccine were burning at the injection site, joint pain and fever, pain at the injection site, muscle pain, and swelling at the injection site. Additionally, the Pfizer vaccine frequently caused minor self-limiting side effects. Moreover, the first dosage was associated with more side effects than the second dosage. Consequently, it is recommended to promote efforts to circulate accurate information about Pfizer vaccine safety and to enhance the monitoring of adverse reactions following vaccination.
